# Coptisine mitigates diabetic nephropathy via repressing the NRLP3 inflammasome

**DOI:** 10.1515/biol-2022-0568

**Published:** 2023-05-11

**Authors:** Jiajia Zhai, Zeping Li, Huifeng Zhang, Zuowei Lu, Yi Zhang, Mo Li, Jian Kang, Zelong Yang, Louyan Ma, Li Ma, Zhengquan Ma, Xiaorui Ma, Fanghong Zhao, Xiaoqing Ma, Yuan Gao, Yuanyuan Zhang, Xiaomiao Li

**Affiliations:** Department of General Practice, Xi’an Ninth Hospital, Xi’an, China; Department of Clinical Medicine, School of Queen Mary, Nanchang University, Nanchang, China; Department of Neurology, Xi’an Electric Power Central Hospital, Xi’an, China; Department of Endocrinology, Xijing Hospital, Air Force Medical University, 127 West Changle Road, Xi’an 710032, China; Department of Microbiology and Pathogen Biology, Basic Medical School, Air Force Medical University, Xi’an, China; Department of Hepatobiliary Surgery, Xijing Hospital, Air Force Medical University, Xi’an, China

**Keywords:** coptisine, diabetic nephropathy, fibrosis, NRLP3 inflammasome

## Abstract

Diabetic nephropathy is a microvascular complication of diabetes mellitus, threatening the health of millions of people. Herein, we explored a blood glucose independent function of coptisine on diabetic nephropathy. A diabetic rat model was established by intraperitoneal administration of streptozotocin (65 mg/kg). Coptisine treatment (50 mg/kg/day) retarded body weight loss and reduced blood glucose. On the other hand, coptisine treatment also decreased kidney weight and the levels of urinary albumin, serum creatinine, and blood urea nitrogen, indicating an improvement of renal function. Treatment with coptisine also mitigated renal fibrosis, with alleviative collagen deposition. Likewise, *in vitro* study showed that coptisine treatment decreased apoptosis and fibrosis markers in HK-2 cells treated with high glucose. Furthermore, after coptisine treatment, the activation of NOD-like receptor pyrin domain containing protein 3 (NRLP3) inflammasome was repressed, with decreased levels of NLRP3, cleaved caspase-1, interleukin (IL)-1β, and IL-18, indicating that the repression of NRLP3 inflammasome contributed to the effect of coptisine on diabetic nephropathy. In conclusion, this study revealed that coptisine mitigates diabetic nephropathy via repressing the NRLP3 inflammasome. It is indicated that coptisine may have the potential to be used in the diabetic nephropathy treatment.

## 1 Introduction

Diabetic nephropathy, a primary and severe complication of diabetes mellitus [1], is a kind of microvascular complication resulting from the injury of renal microvessels. It is one of the main causes of chronic kidney disease, which leads to the end-stage renal disease [2]. Approximately 40% of the diabetic population develop diabetic nephropathy, which is related to the growing mortality of diabetic population [3] and meanwhile poses a huge burden on the diabetic population as well as the health care system.

Growing evidence agrees that inflammation occupies a vital place in the progression of diabetic nephropathy [4]. Inflammasome is activated in response to microbial invasion or non-microbial dangers (for instance, increased reactive oxygen species and high level of glucose), leading to the stimulation to adaptive immunity, release of proinflammatory factors, or occurrence of cellular apoptosis [5]. Inflammasome is composed of several ingredients, including a pattern recognition receptor (for example, NOD-like receptor pyrin domain containing protein 3 (NRLP3)), an adaptor protein apoptosis-associated speck-like protein, and a caspase recruitment domain. Once activated, NLRP3 inflammasome recruits pro-caspase-1, resulting in a caspase-1-depentent maturation and release of proinflammatory factors, such as interleukin (IL)-1β and IL-18 [6]. Increasing evidence reveals the vital role of NLRP3 inflammasome in the pathogenesis of diabetic nephropathy. NLRP3 inflammasome suppressing may be a promising therapy for the treatment of diabetic nephropathy [5].

Natural medicines are commonly used in the Asia region for the treatment of endocrine and metabolic diseases, tumors, and inflammation. Coptisine (C_19_H_14_NO_4_) is a natural isoquinoline alkaloid existing in *rhizoma coptidis*. It exhibits a variety of functions, including anti-cancer [7,8], anti-inflammation [9], anti-oxidant [10,11], myocardial protection [12,13], and anti-diabetes [14]. Our previous study revealed that coptisine decreased oxidative stress and protected kidney in diabetic rats via the Nrf2 signal [15]. Interestingly, coptisine was also able to decrease blood glucose and increase insulin [15,16]. We wondered whether its effect on diabetic nephropathy was also attributed to its function independent of blood glucose modulation. In the present study, we explored the effect of coptisine on diabetic nephropathy and its underlying mechanism.

## 2 Materials and methods

### 2.1 Animal experimental design

This research was designed to explore the effect of coptisine on diabetic nephropathy. This study was conducted in accordance with the Care and Use of Laboratory Animals and approved by the Xi’an Ninth Hospital Ethics Committee. Healthy male SD rats (180–220 g) were obtained from Changsheng Biotechnology Co., Ltd (Benxi, China), fed in a standard environment (temperature: 21–23°C, humidity: 45–55%, 12 h light/dark cycles), and accessed to food and water freely. Rats were divided into three groups: Con (control), DN (diabetic nephropathy), and DN + Cop (diabetic nephropathy with coptisine treatment) (*n* = 6/group). A streptozotocin-induced rat model of type I diabetes mellitus was used in this study. For rats from the DN and DN + Cop groups, type I diabetes mellitus was induced by streptozotocin (Aladdin Biochemical Technology Co., Ltd, Shanghai, China; 65 mg/kg, intraperitoneal administration) as described in our previous study [15]. Control mice (age-matched) received an equal amount of vehicle intraperitoneally. Rats were regarded as diabetic when the level of blood glucose was higher than 16.7 mM on the 3rd day after streptozotocin administration. After verification, the diabetic rats were treated with coptisine (50 mg/kg/day, intragastric administration) or equal amount of vehicle for 56 consecutive days. After diabetes establishment, the body weight of rats in each group was recorded every 2 weeks and the fasting blood glucose was determined every 4 weeks. On the other hand, 24 h urine samples were harvested and the level of urinary albumin was measured. At the end of this study, the blood was harvested and the rats were sacrificed by drawing blood under deep anesthesia (2–3% isoflurane). The kidney tissues were harvested and renal weight was recorded.


**Ethical approval:** The research related to animal use has been complied with all the relevant national regulations and institutional policies for the care and use of animals and has been approved by the Xi’an Ninth Hospital Ethics Committee.

### 2.2 Determination of urinary albumin, serum creatinine, and blood urea nitrogen

Urine during 24 h was collected by keeping the rats individually in a metabolic cage. The concentration of urinary albumin was determined with a Urinary Albumin Quantitative Determination Kit (Nanjing Jiancheng Bioengineering Institute Co., Ltd, Nanjing, China). Serum creatinine level was determined with a Creatinine Determination Kit (Nanjing Jiancheng Bioengineering Institute Co., Ltd). Blood urea nitrogen level was determined using a Blood Urea Nitrogen Determination Kit (Nanjing Jiancheng Bioengineering Institute Co., Ltd) according to the protocol.

### 2.3 Masson’s trichrome staining

Kidney tissues of rats from each group were fixed in 4% paraformaldehyde, routinely embedded in paraffin and cut into 5-μm sections. Following deparaffinization and rehydration, the sections were subjected to routine Masson’s trichrome staining. Images were captured with a microscope (Olympus Corporation, Tokyo, Japan) at 200× magnification.

### 2.4 Immunohistochemistry

Immunohistochemistry was performed to detect fibronectin level in kidney tissues. The sections were heated in citric acid buffer at 100°C for 10 min for antigen retrieval. Thereafter, the sections were incubated with 3% H_2_O_2_ for 15 min to inactivate endogenous peroxidases and blocked in 1% bovine serum albumin (Sangon Biotech, Shanghai, China), followed by incubation with primary antibody against fibronectin (1:200; Affinity Biosciences, Changzhou, China) at 4°C overnight. Then, the sections were incubated with horseradish peroxidase (HRP)-labeled secondary antibodies (1:500; ThermoFisher Co., Ltd, Waltham, MA, USA) at 37°C for 60 min. Thereafter, the sections were developed using a DAB kit (Maixin Biotechnology Co., LTD, Fuzhou, China) and counterstained with hematoxylin. Images were captured with a microscope at 400× magnification.

### 2.5 Western blot

Kidney tissues of rats from each group were harvested. Proteins in kidney tissues and cells were extracted using radioimmunoprecipitation assay lysis buffer (Solarbio, Beijing, China) containing 1% phenylmethanesulfonyl fluoride (Solarbio) on ice. After determination of protein concentration with a BCA Assay Kit (Solarbio), the total protein lysates were separated by sodium dodecyl sulfate polyacrylamide gel electrophoresis and then transferred onto polyvinylidenedifluoride (PVDF) membranes (Millipore Corp, Darmstadt, Germany). Nonspecific binding sites were blocked with 5% skim milk. Then, the PVDF membranes were incubated overnight with the following primary antibodies: fibronectin (1:500; Affinity Biosciences), α-smooth muscle actin (α-SMA) (1:500; Affinity Biosciences), collagen I (1:1,000; Affinity Biosciences), collagen III (1:1,000; Affinity Biosciences), NLRP3 (1:500; Affinity Biosciences), cleaved caspase-1 (1:1,000; Affinity Biosciences), and GAPDH (1:5,000; Solarbio Life Science) at 4°C, followed by incubation at 37°C for 60 min with HRP-conjugated secondary antibodies (1:3,000; Solarbio). The membranes were visualized using an Enhanced Chemiluminescent Substrate Kit (Solarbio).

### 2.6 Immunofluorescence

Immunofluorescence was performed to detect the level of NLRP3 in kidney tissues. After antigen retrieval, the sections were blocked with 1% bovine serum albumin for 15 min, followed by incubation with primary antibody against NLRP3 (1:200; Affinity Biosciences) at 4°C overnight. Then, the sections were incubated with Cy3-labeled secondary antibodies (1:200; Invitrogen, ThermoFisher Co., Ltd) for 60 min. After counterstaining with DAPI, the sections were observed under a fluorescence microscope (Olympus) and images were captured at 400× magnification.

For fibronectin detection in HK-2 cells through immunofluorescence, HK-2 cells were seeded onto glass slides and then subjected to indicated treatments. The cells were fixed with 4% paraformaldehyde and permeabilized with 0.1% tritonX-100. After blockade with 1% bovine serum albumin, the cells were incubated with primary antibody against fibronectin (1:200, Affinity Biosciences) at 4°C overnight. Thereafter, Cy3-labelled secondary antibody (1:200, Invitrogen) was added onto cells and incubated for 60 min at room temperature. Cell nuclei were stained with DAPI (Aladdin Biochemical Technology Co., Ltd). The cells were observed under a fluorescence microscope and images were captured at 400× magnification.

### 2.7 Enzyme-linked immunosorbent assay (ELISA)

The supernatants of cell medium and kidney tissue homogenates were used to determine IL-1β and IL-18 levels using corresponding ELISA kits (Fine Test, Wuhan, China; MultiSciences Biotechnology, Hangzhou, China). The absorbance was measured at 450 nm with a microplate reader. The concentrations of IL-1β and IL-18 were calculated based on the standard curve according to the instructions.

### 2.8 Cell treatment

HK-2 cells were obtained from Procell (Wuhan, China). Cells were grown in minimum Eagle’s medium (Solarbio Life Science) supplementary with 10% fetal bovine serum (Procell, Wuhan, China) at 37°C, in a humidified atmosphere with 5% CO_2_. For coptisine treatment, the cells were treated with indicated concentrations of coptisine (Aladdin Biochemical Technology Co., Ltd), with/without high-glucose stimulation for 48 h.

### 2.9 3-(4,5-Dimethyl-2-thiazolyl)-2,5-diphenyl-2-*H*-tetrazolium bromide (MTT) assay

The cells were seeded into a 96-well plate (4 × 10^3^ cells/well). After indicated treatment for 48 h, MTT (Beyotime Institute of Biotechnology, Shanghai, China) was employed to determine the cell viability according to the protocol.

### 2.10 Apoptosis determination

After indicated treatment, the cells were stained with an Apoptosis Determination Kit (KeyGen Biotechnology Co., Ltd, Nanjing, China) according to the manufacturer’s protocol. Thereafter, the cells were detected using a flow cytometer (NovoCyte, ACEA Biosciences, Inc, Santiago, CA, USA).

### 2.11 Statistical analyses

Data were shown as means ± standard deviation (SD). The one-way or two-way analysis of variance (with Tukey’s multiple comparison test as the post hoc) was employed to compare the discrepancies among groups. *p* < 0.05 indicates a significant difference.

## 3 Results

### 3.1 Coptisine improves renal function in diabetic rats

**Figure 1:**
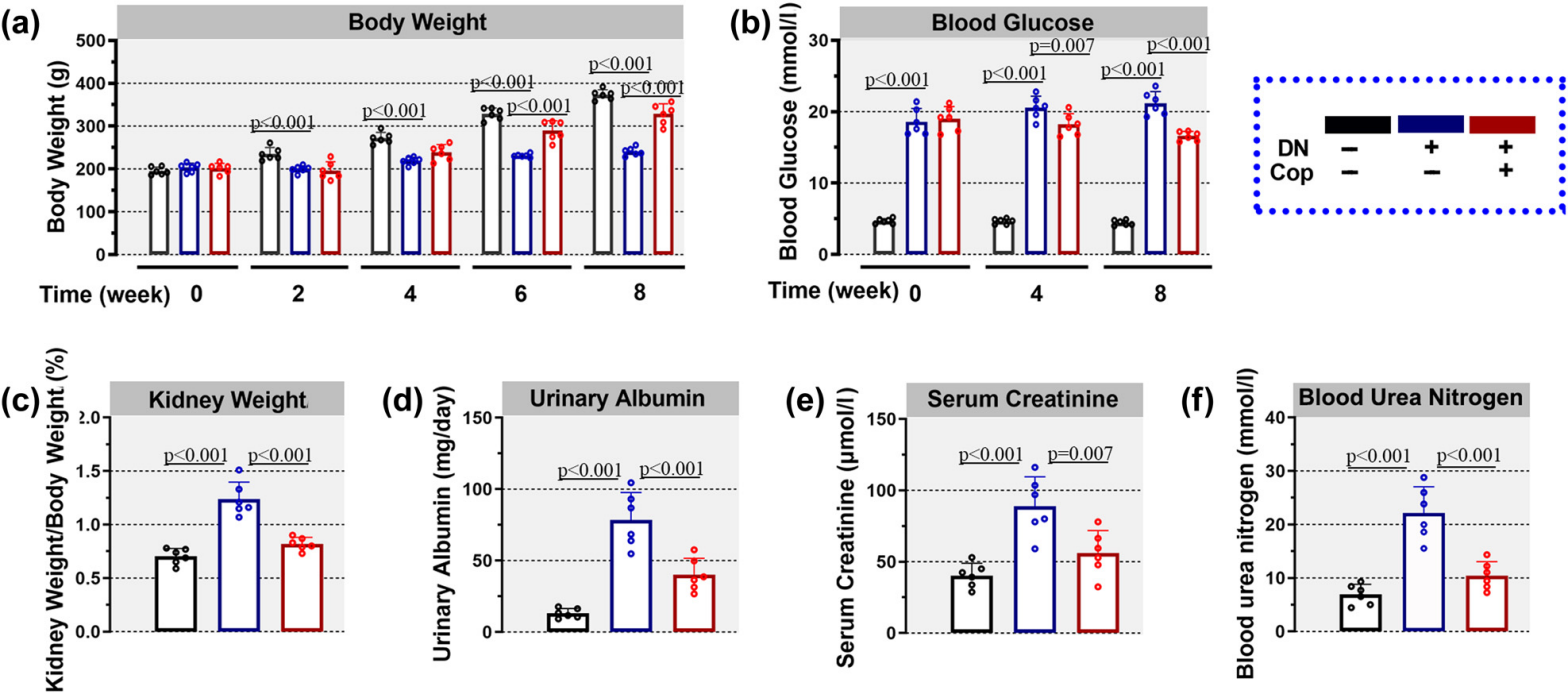
Coptisine improves the renal function of diabetic rats. After treatment with coptisine, the body weight of rats was recorded every 2 weeks (a) and the level of blood glucose was recorded every 4 weeks (b). The kidney weight (c), urine protein level (d), serum creatinine level (e), and blood urea nitrogen (f) were determined after coptisine treatment. *N* = 6 for each experiment. * *p* < 0.05. Cop, coptisine; DN, diabetic nephropathy.

To investigate the effect of coptisine on diabetic nephropathy, the rats received coptisine treatment for 56 consecutive days. Thereafter, their body weight and blood glucose were recorded. Rats in the DN group showed a lower gain of body weight than the Con group (Figure 1a). However, upon treatment with coptisine, the gain of body weight of rats in the DN + Cop group was much quicker than the DN group (Figure 1a). After establishment of diabetic model, the blood glucose of rats in the DN group was much higher than the control rats, whereas the blood glucose was reduced by coptisine treatment at weeks 4 and 8 (Figure 1b). It is demonstrated that coptisine retarded body weight loss and blood glucose ascendance induced by diabetes.

Regarding the effect of coptisine on renal functions, the kidney weight, urinary albumin, serum creatinine, and blood urea nitrogen were determined. The kidney weight of rats in the DN group was much higher than the control group, which was later suppressed by coptisine treatment (Figure 1c). Also, the urinary albumin, serum creatinine, and blood urea nitrogen, which were increased in the diabetic rats, were diminished upon coptisine treatment (Figure 1d–f). This study suggested that coptisine improved the renal function of diabetic rats.

### 3.2 Coptisine alleviates renal fibrosis in diabetic rats

**Figure 2:**
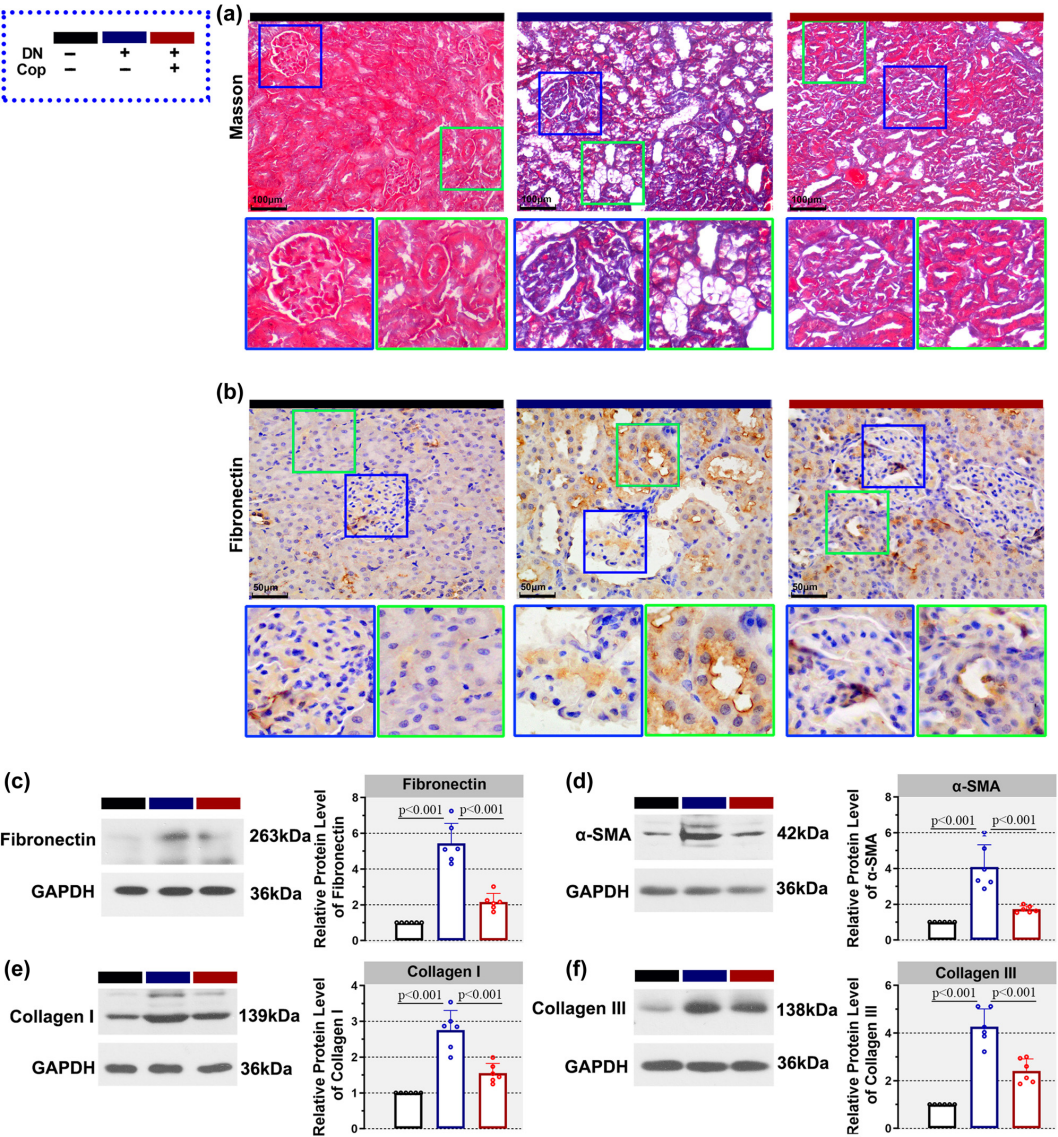
Coptisine alleviates renal fibrosis in diabetic rats. Upon treatment with coptisine, the collagen deposition was determined by Masson’s trichrome staining ((a), 200×, scale bar = 100 μm) and the level of fibronectin in the kidney tissues of rats was detected by immunohistochemistry ((b), 400×, scale bar = 50 μm). Besides, western blot was also conducted to determine fibronectin (c), α-SMA (d), collagen I (e), and collagen III (f) in the kidney tissues. *N* = 6 for each experiment. *p < 0.05. Cop, coptisine; DN, diabetic nephropathy.

Fibrosis is a crucial pathological characteristic of diabetic nephropathy. The effect of coptisine on renal fibrosis was determined by Masson’s trichrome staining. Compared with the Con group, rats in the DN group showed increased collagen deposition. Later, treatment with coptisine reduced collagen deposition in rats in the DN + Cop group, both in glomerulus and uriniferous tubules (Figure 2a). In addition, renal fibrosis was evaluated by immunohistochemistry with firbonectin antibody. Increased fibronectin-positive cells were observed in the kidneys of diabetic rats compared with the control rats, which were later declined by coptisine treatment (Figure 2b). Otherwise, the levels of fibronectin, α-SMA, collagen I, and collagen III, which are markers of fibrosis, were detected by western blot. Compared with the Con group, there were upheavals in all these four protein levels in the kidney tissues of rats from the DN group. Consistent with our aforementioned results, upon treatment with coptisine, the upheaval levels of fibronectin, α-SMA, collagen I, and collagen III were declined (Figure 2c–f). These results illustrated that coptisine alleviated renal fibrosis of diabetic nephropathy.

### 3.3 Coptisine represses the NLRP3 inflammasome in rats with diabetic nephropathy

**Figure 3:**
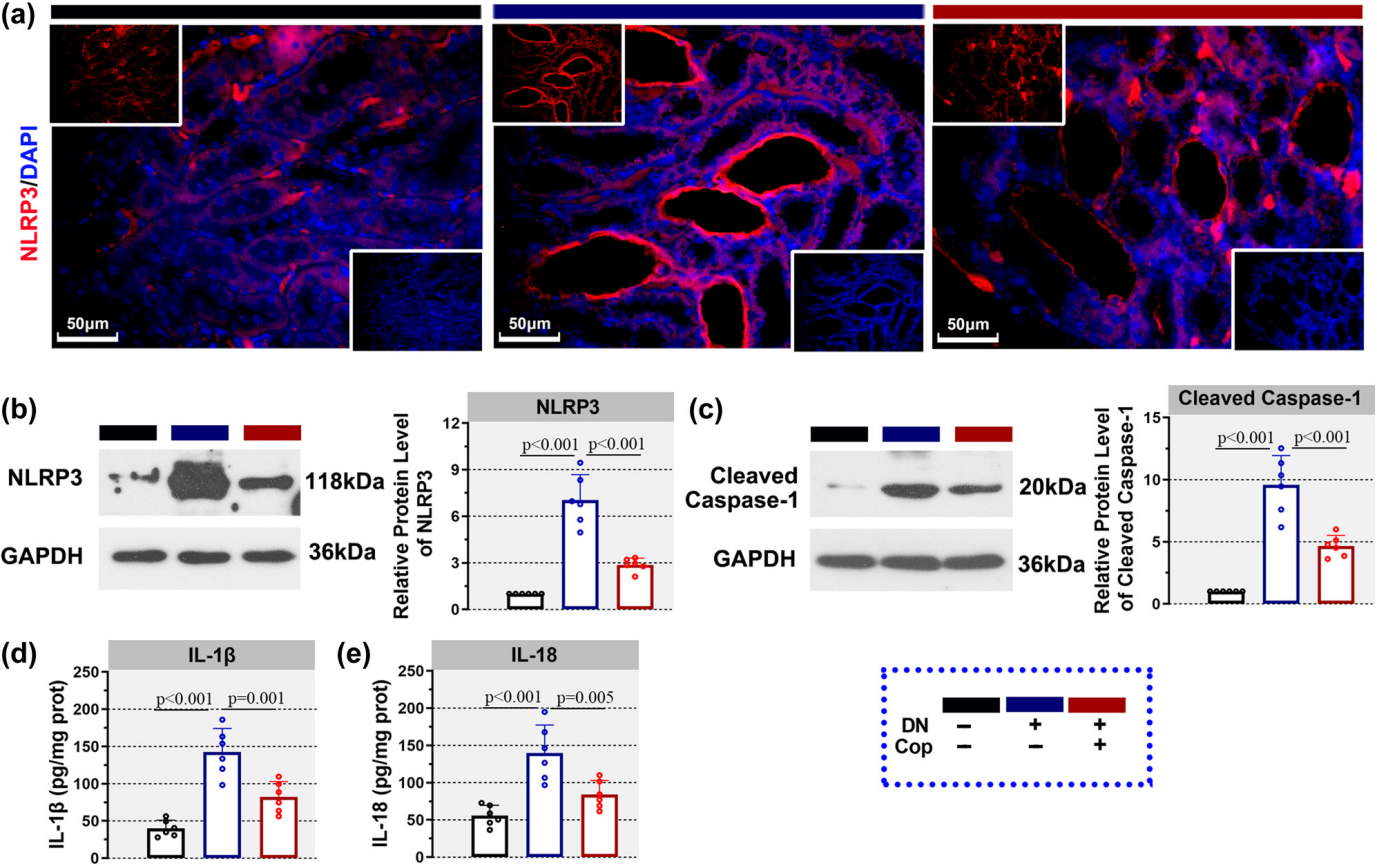
Coptisine represses the activation of NLRP3 inflammasome in diabetic rats. The level of NLRP3 in kidney was determined by immunofluorescence ((a), 400×, scale bar = 50 μm). Red fluorescence indicated NLRP3, and blue fluorescence indicated DAPI. Western blot was also conducted to determine NLRP3 (b) and cleaved caspase-1 (c). The release of IL-1β (d) and IL-18 (e) was monitored by ELISA. *N* = 6 for each experiment. *p < 0.05. Cop, coptisine; DN, diabetic nephropathy; ELISA, enzyme-linked immunosorbent assay.

NLRP3 inflammasome relates to the onset of diabetic nephropathy. To further explore whether the effect of coptisine on diabetic nephropathy is associated with the activation of NLRP3 inflammasome, the level of NLRP3, an important component of the NLRP3 inflammasome, was detected by immunofluorescence as well as western blot after treatment with coptisine. In the kidneys of rats from the DN group, there was more NLRP3 expression than the Con group, whereas, after treatment with coptisine, the augmented NLRP3 was decreased (Figure 3a). Western blot assay showed similar results. The raised NLRP3 level in the kidney tissues of rats from the DN group was diminished by coptisine treatment (Figure 3b). Also, the increased cleaved caspase-1 level in the DN group was also declined upon coptisine treatment (Figure 3c). Moreover, IL-1β and IL-18, which were elevated in the kidneys of rats from the DN group, were reduced after treatment with coptisine (Figure 3d and e). These results indicated that coptisine suppressed the activation of NLRP3 inflammasome in the kidneys of rats with diabetic nephropathy.

### 3.4 Coptisine diminished apoptosis induced by high glucose

**Figure 4:**
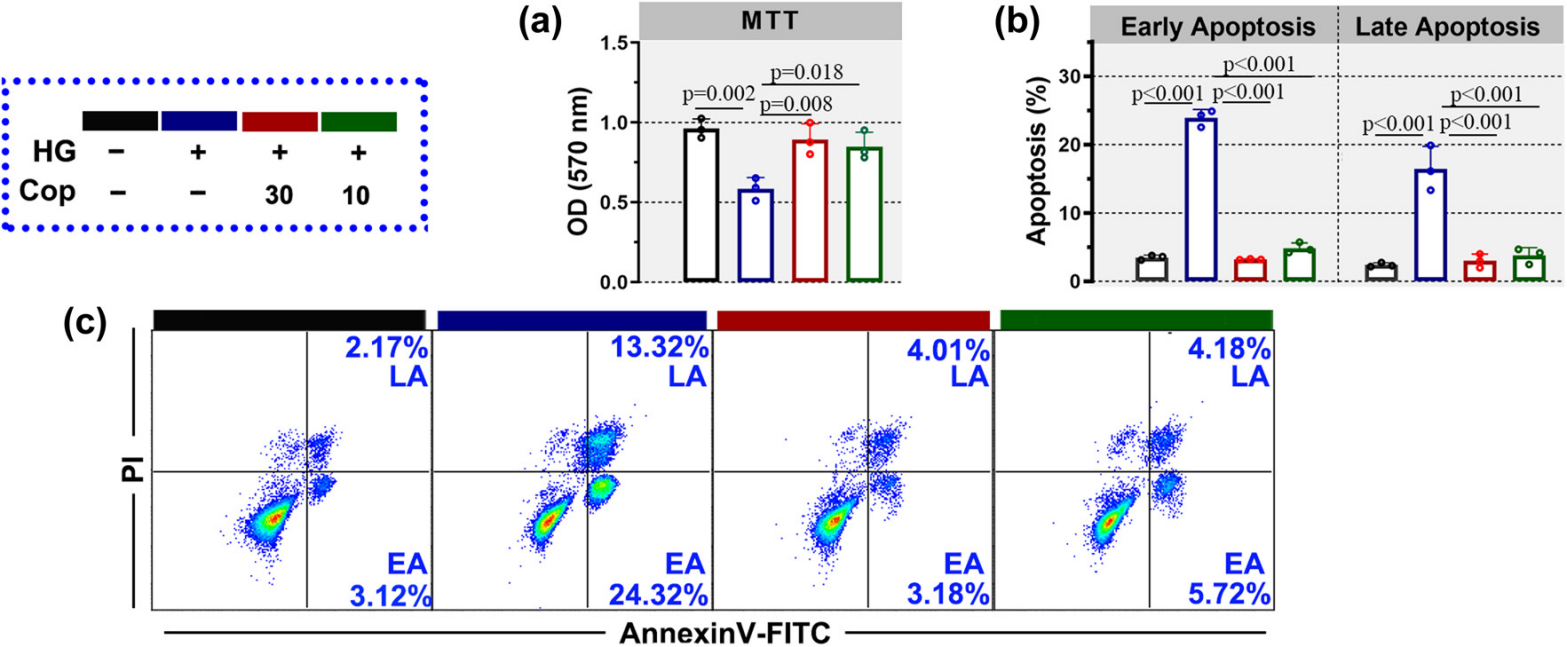
Coptisine decreased apoptosis induced by high glucose. HK-2 cells were treated with high glucose and coptisine. Thereafter, the cell viability was determined by MTT (a). The cellular apoptosis was detected by flow cytometry (b and c). *N* = 3 for each experiment. *p < 0.05. Cop, coptisine; DN, diabetic nephropathy; EA, early apoptosis; LA, late apoptosis.

**Figure A1:**
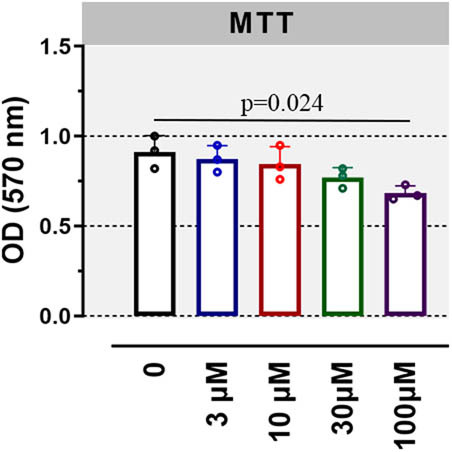
The effect of coptisine on HK-2 cells. After treatment with different concentrations of coptisine (3–100 μM), the cell viability was determined by MTT. * *p* < 0.05.

HK-2 cells cultured in high-glucose environment were usually employed as the *in vitro* cell model of diabetic nephropathy [16]. Before investigating the effect of coptisine on this cell model, the toxicity of coptisine was determined by MTT. Coptisine at 0–30 μM showed no remarked toxicity on HK-2 cells (Figure A1). Thereafter, HK-2 cells were treated with/without 10 or 30 μM coptisine and high glucose. The cell viability of HK-2 was injured by high glucose, which was later elevated by 10 or 30 μM coptisine (Figure 4a). Also, apoptosis (including early apoptosis and late apoptosis) induced by high glucose was repressed by 10 or 30 μM coptisine (Figure 4b and c). These results indicated that coptisine repressed cellular apoptosis induced by high glucose.

### 3.5 Coptisine reduced the levels of fibrosis markers and suppressed the activation of NLRP3 inflammasome *in vitro*

**Figure 5:**
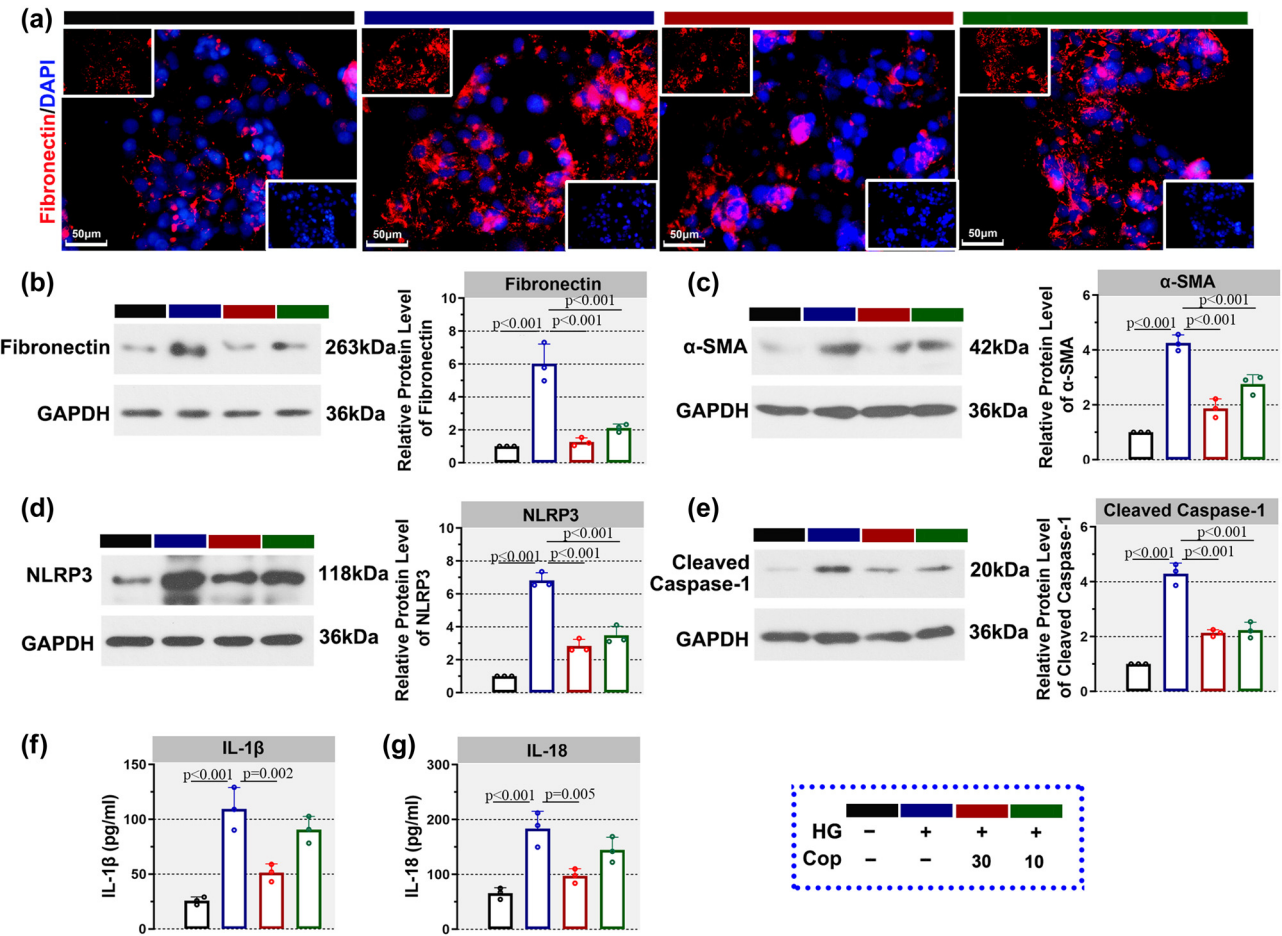
Coptisine reduced the levels of fibrosis markers and suppressed the activation of NLRP3 inflammasome in HK-2 cells. The level of fibronectin in HK-2 cells treated with high glucose and coptisine was determined by immunofluorescence ((a), 400×, scale bar = 50 μm). Red fluorescence indicated fibronectin, and blue fluorescence indicated DAPI. Western blot was also conducted to determine fibronectin (b), α-SMA (c), NLRP3 (d), and cleaved caspase-1 (e). In addition, ELISA was performed to detect the levels of IL-1β (f) and IL-18 (g) in the cell medium. *N* = 3 for each experiment.* *p* < 0.05. Cop, coptisine; HG, high glucose, ELISA, enzyme-linked immunosorbent assay.

Consistent with the aforementioned results, the level of fibronectin in HK-2 cells treated with high glucose was increased, while treatment with coptisine at 30 or 10 μM reduced the expression of fibronectin (Figure 5a). As evidenced by western blot, the levels of fibronectin and α-SMA were augmented in HK-2 cells treated with high glucose while later declined by treatment with coptisine (Figure 5b and c). In addition, in HK-2 cells treated with high glucose, the elevated levels of NLRP3 and cleaved caspase-1, which indicates the activation of NLRP3 inflammasome, were declined by coptisine treatment (Figure 5d and e). Similarly, the increased IL-1β and IL-18 levels in HK-2 cells treated with high glucose were diminished by 30 μM coptisine (Figure 5f and g). These results revealed that coptisine repressed fibrosis and the activation of NLRP3 inflammasome in HK-2 cells treated with high glucose.

## 4 Discussion

In the current clinical treatment of diabetic nephropathy, strict blood glucose and hypertension control is the main routines. However, the consequence is unsatisfactory. Thus, there is an urgent need to find a promising drug for the treatment of diabetic nephropathy. In the present study, we explored the protective effect of coptisine against diabetic nephropathy. We found that coptisine repressed renal fibrosis induced by diabetes through suppressing the NLRP3 inflammasome, indicating that coptisine may become a promising drug for the treatment of diabetic nephropathy.

In the present study, we showed that coptisine mitigated diabetic nephropathy. Hyperglycemia acts as a main cause of renal injury during diabetes mellitus, which contributes to the pathogenesis of diabetic nephropathy. This study and our previous study showed that coptisine decreased blood glucose, which is beneficial to control diabetes. Our previous study showed that coptisine also decreased blood lipid and increased insulin [15]. As coptisine can decrease blood glucose through improving glucose metabolism via mitochondrial energy generation [16], we wondered whether coptisine mitigated diabetic nephropathy through modulating glucose metabolism or in a glucose metabolism-independent manner. Herein, we revealed that coptisine performed its protective effect on renal tubular cell injury via repressing inflammasome activation, a glucose metabolism-independent manner. Zhou et al. also showed that coptisine exhibited a potential to treat diabetic vasculopathy [11], which provided additional evidence for the application of coptisine in diabetic nephropathy treatment. Shi et al. showed that coptisine did not show cytotoxicity in short-term treatment [16]. Our study also showed that treatment with coptisine for 8 weeks showed renal protective effect. However, there is still a need to illustrate the long-term effect of coptisine before its usage in clinic.

Renal fibrosis is regarded as a crucial indicator reflecting the kidney function deterioration. It is a common pathological progression in several renal diseases, including diabetic nephropathy, mainly attributed to the accumulation of extracellular matrix and mesangial expansion [17]. These hallmarks result in irreversible injury to renal functions. Renal fibrosis is regarded as a vital final process of diabetic nephropathy to kidney failure [18]. It is reported that inhibiting renal fibrosis benefits to the therapy of diabetic nephropathy [19,20]. In this study, besides the effects on blood glucose, coptisine mitigated renal fibrosis induced by diabetes mellitus, which was evidenced by less collagen deposition and fibrosismarkers. Thus, we speculated that suppression of renal fibrosis may contribute to the protective effect of coptisine on diabetic nephropathy. Renal tubular cells play a critical role in renal fibrosis. In this study, the increased level of fibrosis markers in renal tubular cell HK-2 induced by high glucose was repressed by coptisine treatment, which provided additional evidence for our hypothesis that coptisine mitigated renal fibrosis during diabetic nephropathy.

The NLRP3 inflammasome exhibits a critical role in diabetic nephropathy pathogenesis. It is involved in the onset of diabetic nephropathy and participates in renal fibrosis [21]. Repressing the NLRP3 inflammasome relieves inflammation, alleviates interstitial fibrosis, and improves renal function [22]. In the present study, we found that coptisine repressed the activation of NLRP3 inflammasome, which may contribute to the effect of coptisine on diabetic nephropathy. In addition, hyperglycemia is able to trigger the NLRP3 inflammmasome [21]. Activation of NLRP3 inflammasome in renal tubular cell HK-2 treated with high glucose was also decreased upon coptisine treatment, which was consistent with the effect of coptisine *in vivo*. Wu et al. also showed that blocking the NLRP3 inflammasome contributed to the function of coptisine in macrophages [23]. This provides additional evidence for our hypothesis that repressing NLRP3 inflammasome may participate in the function of coptisine on diabetic nephropathy.

Repression of NLRP3 inflammasome ameliorates renal fibrosis via a variety of mechanisms, such as improving endoplasmic reticulum stress and mitochondrial dysfunction and declining inflammation and oxidative stress [22,24,25]. In our previous study, we found that the Nrf2 signal, a famous anti-oxidant signal, also contributed to the function of coptisine [15]. Interestingly, inhibiting the Nrf2 signal results in NLRP3 inflammasome activation [26]. It is speculated that coptisine suppressed the activation of NLRP3 inflammasome, maybe through the Nrf2 signal. On the other hand, coptisine also suppressed the activation of NF-κB pathway, which modulated the expression of NLRP3 [27,28]. Thus, we speculated that the NF-κB pathway may also attribute to NLRP3 inflammasome suppression by coptisine in diabetic nephropathy. As a limitation, in the present study, we did not explore how coptisine directly suppressed the activation of NLRP3 inflammasome. Fortunately, Wu et al. pointed out that coptisine entered into the active sites of caspase-1 and interacted with caspase-1 via hydrophobic bonding or forming hydrogen bonds with the Gln283 residue of caspase-1, thus influencing the bond of pro-caspase-1 to apoptosis-associated speck-like protein and caspase recruitment domain and attenuating the secretion of mature IL-1β in RAW264.7 [23]. This may be applicable to the function of coptisine on NLRP3 inflammasome in this study. Hence, the repression of NLRP3 inflammasome contributes to the function of coptisine on diabetic nephropathy, maybe through interacting with caspase-1 directly or modulating the Nrf2 or NF-κB pathways indirectly. However, further evidence is still needed.

## 5 Conclusion

In conclusion, in the present study, we revealed that coptisine remitted diabetic nephropathy via suppressing the activation of NLRP3 inflammasome. It is indicated that coptisine has the potential to be used in the treatment of diabetic nephropathy.
